# Artificial Intelligence for Exosomal Biomarker Discovery for Cardiovascular Diseases: Multi-Omics Integration, Reproducibility, and Translational Prospects

**DOI:** 10.3390/cells15030304

**Published:** 2026-02-05

**Authors:** Rasit Dinc, Nurittin Ardic

**Affiliations:** 1 INVAMED Medical Innovation Institute, New York, NY 10007, USA; 2 Med-International UK Health Agency Ltd., Warwickshire CV11 6LT, UK; nurittinardic@yahoo.com

**Keywords:** extracellular vesicles, exosomes, cardiovascular disease, biomarkers, artificial intelligence, multi-omics

## Abstract

Exosomes and other extracellular vesicles (EVs) carry microRNAs, proteins, and lipids that reflect cardiovascular pathophysiology and can enable minimally invasive biomarker discovery. However, EV datasets are highly dimensional and heterogeneous, strongly influenced by pre-analytic variables and non-standardized isolation/characterization workflows, limiting reproducibility across studies. Artificial intelligence (AI), including machine learning (ML), deep learning (DL), and network-based approaches, can support EV biomarker development by integrating multi-omics profiles with clinical metadata. These approaches enable feature selection, disease subtyping, and interpretable model development. Among the AI approaches evaluated, ensemble methods (Random Forest, gradient boosting) demonstrate the most consistent performance for EV biomarker classification (AUC 0.80–0.92), while graph neural networks (GNNs) are particularly promising for path integration but require larger validation cohorts. Evolutionary neural networks applied to EV morphological features yield comparable discrimination but face interpretability challenges for clinical use. Current studies report promising discrimination performance for selected EV-derived panels in acute myocardial infarction and heart failure. However, most evidence remains exploratory, based on small cohorts (*n* < 50) and limited external validation. For clinical implementation, EV biomarkers need direct comparison against established standards (high-sensitivity troponin and natriuretic peptides), supported by locked-in assay plans, and validation in multicenter cohorts using MISEV-aligned protocols and transparent AI reporting practices. Through a comprehensive, integrative, and comparative analysis of AI methodologies for EV biomarker discovery, together with explicit criteria for reproducibility and translational readiness, this review establishes a practical framework to advance exosomal diagnostics from exploratory research toward clinical implementation.

## 1. Introduction

Cardiovascular diseases (CVDs) remain the leading cause of death, accounting for an estimated 17.3 million deaths worldwide each year [1]. In addition to their high prevalence, CVDs constitute a significant global health problem with profound economic and social consequences [2]. Although conventional pharmacotherapy and surgery can alleviate symptoms and reduce mortality, there is still a lack of effective clinical strategies to prevent the catastrophic development of CVDs, including repairing damaged myocardium or halting the progression to heart failure (HF) after myocardial infarction (MI) [3].

Biomarker detection is critical for the early diagnosis and management of CVDs, enabling timely treatment and improved outcomes [4,5]. Traditional biomarkers such as cardiac troponins, natriuretic peptides (BNP/NT-proBNP), and soluble ST2 are well established in clinical practice, but their sensitivity and specificity are limited, particularly in subclinical or regenerative settings [6]. This highlights the need for more sensitive, minimally invasive, and dynamic biomarkers that can better capture disease onset, progression, and prognosis.

Inflammation has been widely recognized as a critical contributing factor to CVDs [7]. In addition to inflammation, there are other underlying molecular mechanisms that have not yet been fully elucidated. In this context, extracellular vesicles (EVs), particularly exosomes, are known to contain a wide variety of biomolecules, including proteins, lipids, nucleic acids, and metabolites, and thus can play important roles in numerous biological functions [8]. Recent evidence confirms their role in myocardial infarction, ischemia–reperfusion injury, atherosclerosis, and heart failure, affecting inflammation, fibrosis, and tissue repair in these diseases [4,8,9]. Their natural abundance and relative stability make exosomes attractive, minimally invasive biomarkers, apart from being potential therapeutic targets [10,11].

The complexity of exosomal cargo, encompassing a variety of RNAs, proteins, lipids, and metabolites, produces high-dimensional, heterogeneous datasets that defy traditional statistical analyses. The relationships between exosomal signatures and cardiovascular outcomes are often subtle and nonlinear, making robust biomarker discovery challenging. Artificial intelligence (AI), particularly machine learning (ML) and deep learning (DL), offers powerful tools to overcome these hurdles [12,13,14]. For example, ML models have demonstrated higher AUCs (e.g., 0.865 versus 0.765) in outperforming traditional risk scores in CVD prediction [15]. DL methods have improved the analysis of extracellular vesicle datasets and increased the accuracy of early disease detection [16]; in CVD contexts, early applications likewise show promising performance on patient-derived EV datasets [12,13]. As precision cardiology evolves, AI-powered analyses are expected to accelerate exosome-based biomarker discovery and optimize their translation into precision cardiology by enabling automated feature extraction, pattern recognition, and integration of multi-omics with clinical data, while maintaining interpretability [17,18].

The convergence of exosomal biology and AI moves beyond traditional single-biomarker approaches in cardiovascular medicine and enables multidimensional analyses that can capture the complexity of disease mechanisms. This integration is particularly timely given recent advances in both fields. Advanced exosome isolation and characterization technologies have improved data quality [8,11]. On the other hand, groundbreaking AI algorithms have demonstrated unprecedented pattern recognition capabilities in biological datasets [12,13]. This synergy creates opportunities for the discovery of novel exosome-based biomarkers that have the potential to transform cardiovascular risk assessment, early diagnosis, and personalized treatment strategies [4,19].

Yet, translating AI-enabled exosomal insights into clinical practice requires overcoming significant challenges. These include standardizing exosome isolation and analytical workflows across laboratories, validating AI models in large and diverse populations, and aligning with regulatory and ethical frameworks [1,15,17,18]. Integration into healthcare systems will also depend on explainable AI solutions that build trust between clinicians and patients. Therefore, acknowledging both opportunities and limitations is crucial to realizing the promise of AI-assisted exosomal biomarker discovery to advance cardiovascular precision medicine.

This narrative review aims to synthesize current developments at the intersection of exosome biology and AI, focusing specifically on diagnostic and therapeutic opportunities in cardiovascular diseases. We highlight the biological roles and biomarker potential of exosomes, review AI approaches for biomarker discovery and classification, and discuss therapeutic applications. We also address translational challenges and present future directions for the integration of AI-enabled exosomal insights into cardiovascular precision medicine.

This review makes four distinct contributions beyond the existing EV–cardiovascular literature: (1) a structured comparative analysis of AI architectures, including supervised ML, DL, and graph neural networks (GNNs), and a critical appraisal of reported performance trends across cardiovascular conditions; (2) a focused examination of the “small *n*, large *p*” problem and recurrent AI overfitting models in current exosomal biomarker studies; (3) a reproducibility-oriented framework integrating MISEV2023 recommendations with transparent AI reporting standards such as TRIPOD and PROBAST; and (4) a translation decision matrix that separates exploratory findings from clinically validated evidence with regulatory pathway-oriented considerations. Rather than cataloging candidate biomarkers or summarizing biological mechanisms, this review highlights how AI methodologies can address analytical challenges unique to high-dimensional EV datasets and support clinically meaningful translation.

This narrative review was informed by searches in PubMed and Scopus up to December 2025 using combinations of keywords related to extracellular vesicles/exosomes (“extracellular vesicles”, “exosomes”, “small EV”), cardiovascular disease (“myocardial infarction”, “heart failure”, “atherosclerosis”, “cardiomyopathy”), and artificial intelligence (“machine learning”, “deep learning”, “multiple omics integration”, “graph neural networks”, “explicit AI”). Where possible, we prioritized studies involving human cohorts, conforming to the MISEV guidelines, demonstrating explicit EV characterization, multiple omics profiling, and/or integration with clinical metadata and validation practices (internal cross-validation, retention testing, temporal validation, or external validation). Basic methodology articles, databases, and reporting guidelines were included to support reproducibility and translational interpretation.

## 2. An Overview of Extracellular Vesicles, Focusing on Exosomes

EVs are lipid bilayer particles secreted by nearly all cell types and found in body fluids. They are increasingly recognized as essential mediators of intercellular communication in both physiological and pathological conditions, including cardiovascular diseases (CVDs). Among EVs, exosomes are of particular interest due to their unique endosomal origin and rich molecular cargo. However, exosomes coexist with other EV subtypes, particularly ectosomes (microvesicles) and apoptotic bodies, necessitating precise classification for reproducibility and interpretation [20,21].

### 2.1. Biogenesis, Classification, and Subtypes of EVs

Particularly in complex biological fluids such as blood, there is no consensus on subtype-specific markers that can reliably distinguish EVs according to their biogenesis (e.g., endosomal-derived "exosomes" versus plasma membrane-derived "ectosomes/microvesicles"). EVs are generally classified as (i) small EVs (mostly including exosome-enriched preparations; ~30–150 nm), (ii) medium/large EVs (mostly including ectosome-/microvesicle-enriched preparations; ~100–1000 nm), and (iii) apoptotic bodies (> 500 nm); this classification acknowledges that size ranges overlap, and cargo composition may vary depending on the isolation method and cellular context. Since biogenesis cannot be precisely determined in most clinical samples and mixed EV populations are frequently isolated together, consensus guidelines recommend operational definitions (e.g., “EVs < 200 nm” or “medium/large EVs > 200 nm”) along with transparent reporting of enrichment and characterization methods (MISEV2018) [20,21,22]. This heterogeneity and methodological variability directly impact downstream multi-omics profiling and contribute to batch effects and reproducibility challenges that need to be addressed in AI-based biomarker discovery processes.

### 2.2. Molecular Cargo, Multi-Omics Complexity, and Analytical Challenges

Exosomes and other EVs carry a diverse molecular load, including proteins, lipids, mRNAs, microRNAs (miRNAs), long non-coding RNAs (lncRNAs), circular RNAs (circRNAs), and metabolites. EV load reflects the physiological or pathological state of the host cell and can modulate recipient cell function, supporting the rationale for using EVs as circulating biomarkers in cardiovascular disease [23].

Publicly available EV resources further illustrate the breadth of EV-associated molecular profiles. The ExoCarta 2024 repository reports cumulative entries of >13,000 proteins, ~3400 mRNAs, ~10,700 miRNAs, and ~4000 lipid species associated with exosomes/EVs [23,24]. Importantly, these values represent aggregate database catalogs compiled across studies and experimental conditions, rather than quantitative cargo counts within a single vesicle preparation or a universal “EV cargo composition.” Differences in isolation workflows, annotation standards, and validation criteria among contributing studies also mean that database aggregates should be interpreted as upper limit inventories of reported EV-associated molecules, rather than as per-vesicle quantifications [23,24]. However, these repositories highlight the inherently multi-omic and high-dimensional nature of EV datasets compared to conventional single-analyte biomarker approaches. A central analytical challenge is the highly heterogeneous nature of EV cargo at the single-vesicle level. Quantitative analysis by Chevillet et al. has shown that even abundant miRNAs often have an average copy number per vesicle that is significantly lower than one, meaning that most individual exosomes do not carry detectable copies of a particular miRNA [25]. This finding reinforces that clinically useful signal detection typically requires population-level profiling and robust statistical or machine learning frameworks, rather than relying on single-molecule assumptions.

Furthermore, EV-associated biomarker abundance can change rapidly following acute cardiovascular events. For example, Wang et al. showed that plasma miR-208a was initially undetectable but rose within 1 h after coronary occlusion and became detectable within 4 h in most AMI patients [26]. This time-dependent behavior highlights the importance of accounting for sampling windows and temporal dynamics when interpreting EV-associated signatures in cardiovascular contexts [26].

Although multiple individual miRNAs (e.g., miR-1, miR-133a, and miR-208a) have been reported to be increased in AMI [26], studies integrating exosome RNAs, proteins, and lipids concurrently to improve diagnostic or prognostic performance are relatively limited. Recent reviews highlight that EV-associated cargoes from multiple molecular classes can offer value for diagnosis and prognosis across cardiovascular disease phenotypes, promoting multi-omic strategies instead of single-analyte approaches [27,28]. However, multi-omics integration further increases dimensionality and the risk of false discovery when cohort sizes are small; this underscores why rigorous computational modeling, leak-aware validation, and reproducible workflows are necessary for developing clinically meaningful EV biomarkers.

**Figure 1:**
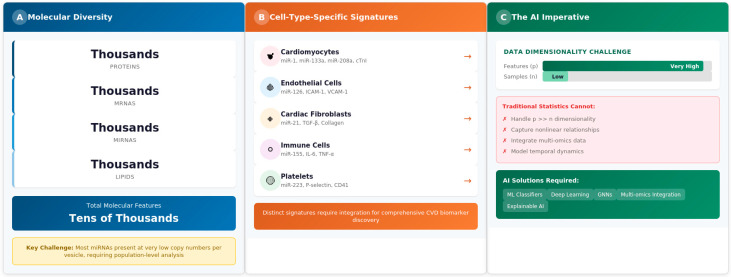
Rationale for exosome complexity and AI-based analysis. (**A**) Molecular diversity showing thousands of cargo types across proteins, mRNAs, miRNAs, and lipids. (**B**) Cell-type-specific exosome signatures in cardiovascular disease. (**C**) Multi-omic data structures motivating AI-based pattern recognition. Detailed methodology is described in Section 2.2. Generated by the authors; information synthesized from publicly available EV sources.

The combination of these challenges generates high-dimensional datasets that exceed the analytical capacity of traditional statistical methods (Figure 1).

In the context of cardiovascular biomarker discovery, the fundamental challenge is not the existence of EV cargo diversity, but how to robustly extract reproducible disease signals from high-dimensional, batch-sensitive multi-omics profiles generated in heterogeneous isolation and characterization workflows. Accordingly, the remainder of this review focuses on practical AI-powered strategies for multi-omics integration, design validation, reproducibility reporting, and translational readiness assessment, rather than providing a comprehensive overview of EV biology.

### 2.3. Methods of Isolation and Characterization

The study of exosomes and other extracellular EVs is greatly influenced by the methods used for their isolation and characterization. Because EVs are heterogeneous in size, density, and cargo composition, methodological choices not only determine the yield and purity of vesicle preparations but also shape subsequent biomarker discovery and translational applications.

#### 2.3.1. Isolation Techniques

Various strategies are used to enrich EVs from plasma, serum, urine, and tissue culture supernatants [20,21,29,30]:

Pre-analytical variability is a significant determinant of EV yield, apparent cargo composition, and downstream biomarker performance, and can easily dominate the “biological signal” in small cohorts. In blood samples, common confounding factors include lipoprotein contamination (HDL/LDL/VLDL and chylomicrons) that can carry abundant RNA and proteins, overlapping with EV size and density, potentially affecting proteomic and small RNA profiling. Hemolysis can artificially inflate candidate signatures and increase intersample noise by releasing erythrocyte-derived miRNAs (e.g., miR-451a and miR-16) and other intracellular components. Platelet activation during venous blood collection or processing (e.g., prolonged tourniquet time, shaking, delayed centrifugation) can significantly increase circulating platelet-derived EVs and alter apparent “disease-related” profiles; anticoagulant choice (EDTA, citrate, or heparin) and processing temperature can further influence EV count and composition. Freeze–thaw cycles and prolonged storage can lead to vesicle rupture, aggregation, or selective cargo degradation, while repeated thawing can increase particle count through fragmentation and create spurious results in nanoparticle tracking analysis. For these reasons, studies should report and harmonize key variables, including collection tubes, processing time, centrifugation protocol, anticoagulant, storage time/temperature, and number of freeze–thaw cycles, and implement hemolysis and lipoprotein quality controls when EV-derived biomarkers are proposed [20,29,30].

Differential ultracentrifugation (UC): UC, the most widely used approach, separates vesicles based on size and density through sequential centrifugation steps. While considered the “gold standard,” it is time-consuming, requires large sample volumes, and often co-purifies protein aggregates and ectosomes.

Size-exclusion chromatography (SEC): SEC separates vesicles from soluble proteins and lipoproteins based on column filtration. It offers higher purity and reproducibility compared to UC, but may result in lower vesicle recovery, especially from low-volume clinical samples.

Precipitation methods: Commercial polymer-based kits provide rapid vesicle isolation by inducing precipitation. While convenient, these methods co-precipitate lipoproteins and protein contaminants, limiting their suitability for quantitative proteomic or lipidomic studies.

Immunoaffinity capture: Antibodies targeting canonical exosome markers (e.g., CD63, CD9, and CD81) can enrich specific vesicle populations. This approach provides high specificity, but at the expense of reduced yield and may bias results toward tetraspanin-enriched vesicles.

Microfluidic-based platforms: Emerging techniques utilize lab-on-a-chip devices that utilize vesicle size, charge, or surface markers for high-throughput isolation. These platforms require minimal sample volume and allow for integration with single-vesicle analysis, but they are not yet standardized for clinical use.

#### 2.3.2. Characterization Techniques

After isolation, EVs must be meticulously characterized to confirm their identity and assess their purity [21,22,31,32,33].

Nanoparticle tracking analysis (NTA): Provides vesicle size distribution and concentration by analyzing Brownian motion. Widely used but limited in resolving particles <50 nm in size or distinguishing exosomes from lipoproteins.

Electron microscopy (TEM, cryo-EM): Provides direct visualization of vesicle morphology and size. While highly informative, it is technically challenging and has low throughput.

Western blotting and flow cytometry: Detects canonical protein markers such as CD9, CD63, CD81, TSG101, and Alix. High-sensitivity flow cytometry approaches are increasingly used to characterize vesicles at the single-particle level.

Omics platforms: Mass spectrometry, RNA sequencing, and lipidomics provide a comprehensive profile of vesicle cargo, essential for biomarker discovery. However, platform-specific biases and limited input material continue to pose challenges.

#### 2.3.3. Standardization and Reproducibility

A persistent obstacle in EV research is the lack of standardization in isolation and characterization workflows. Different techniques enrich for overlapping but distinct vesicle populations, making comparisons between studies difficult. To address this issue, the MISEV2018 guidelines recommend that researchers report operational definitions (e.g., “EVs smaller than 200 nm”) when biogenetic origin cannot be confirmed and require at least three types of characterization evidence (morphology, size distribution, and protein markers) [20]. Recent updates further emphasize the need for transparent reporting of pre-analytical variables such as sample processing, storage, and processing delays, which can significantly impact vesicle integrity [29].

Exosome and EV research are tightly linked to methodological rigor. Each isolation technique offers trade-offs between purity, yield, and scalability, while characterization platforms differ in resolution and efficiency. These methodological challenges contribute to inter-study variability and complicate the translation of EV-based biomarkers into cardiovascular medicine. Recognizing these limitations is critical not only for experimental reproducibility but also for applying AI approaches that rely on high-quality, standardized input data to identify robust biomarker signatures.

### 2.4. Translational Applications: Exosome-Device Integration

Beyond their role as biomarkers, exosomes are increasingly being investigated as therapeutic carriers and bioactive coatings in cardiovascular medicine. Their natural lipid bilayers, rich cargo diversity, and low immunogenicity position them as versatile alternatives to traditional drug delivery platforms.

A pioneering example is the exosome-eluting stent (EES). These stents, engineered with mesenchymal stem cell-derived exosomes, demonstrated accelerated endothelialization, reduced neointimal hyperplasia, and improved vascular repair in preclinical models compared to drug-eluting stents [34]. More recently, platelet membrane-coated exosome-mimicking nanovesicles have been designed to selectively target atherosclerotic plaques. They have enhanced vascular healing by reducing cholesterol efflux and lipid accumulation [35]. Such bioinspired vesicles combine the advantages of exosomal cargo delivery with the targeting capabilities of cell-mimicking coatings. These approaches demonstrate the feasibility of leveraging exosomal biology in device-based cardiovascular therapy.

Although still in the preclinical stage, these studies highlight the theranostic potential of exosomes. Their molecular cargo serves as a source of biomarkers, while their vesicular structure serves as a therapeutic tool. This dual role highlights the importance of continuous methodological improvement. The complexity of optimizing both diagnostic signatures and therapeutic applications creates an ideal application area for AI-driven approaches to optimize both diagnostic and therapeutic applications of exosomes in CVDs.

### 2.5. Molecular Mechanisms of Exosomal Intercellular Communication

The functional importance of exosomes in cardiovascular diseases depends on the complex molecular mechanisms governing their biogenesis, cargo sorting, and cellular uptake. Exosomal biogenesis occurs through two primary mechanisms that determine cargo composition and therapeutic potential. The ESCRT (Endosomal Sorting Complexes Required for Transport) pathway involves the sequential activation of ESCRT-0, -I, -II, and -III complexes; ESCRT-0 recognizes ubiquitinated proteins, while ESCRT-III facilitates vesicle fragmentation [4]. This pathway primarily sequesters stress response proteins and signaling molecules critical to cardiovascular pathophysiology. ESCRT-independent mechanisms utilize microdomains organized by ceramide-induced membrane budding and tetraspanins (CD9, CD63, CD81, and CD82). The ceramide-induced membrane budding pathway is particularly important for the packaging of protective miRNAs such as miR-126 and miR-210. Membrane regulators such as tetraspanins contribute to EV biogenesis and selective cargo partitioning. In vivo studies also indicate that EV integrin repertoires influence tissue uptake and organotropism [36,37]. In cardiovascular regards, CD63 is important for the loading of cardioprotective miRNAs, while CD9 packages anti-inflammatory proteins [4].

Exosomal uptake occurs through endocytosis and receptor-mediated mechanisms. Receptor-mediated uptake involves integrin interactions that determine tissue tropism; integrin α6β4 enhances cardiac uptake, while αvβ3 targets endothelial cells [36,37].

These functional and molecular mechanistic complexities underscore that AI frameworks capable of integrating biogenesis, sorting, and uptake variables offer unprecedented opportunities for developing precision exosomal therapeutics. However, these complexities require standardized protocols and comprehensive characterization to ensure reproducible AI model training and successful clinical translation.

## 3. Exosomes in Cardiovascular Diseases

Exosomes and other extracellular vesicles have increasingly recognized roles in the onset, progression, and recovery from CVDs. Their molecular cargo reflects the activity of cardiomyocytes, endothelial cells, fibroblasts, and immune cells, enabling them to serve as both mediators of intercellular signaling and circulating biomarkers of disease states [38,39]. Recent advances in EV biology have revealed their involvement in processes such as myocardial ischemia–reperfusion injury, adverse ventricular remodeling, atherosclerosis, arrhythmia, and heart failure [3,4].

The clinical significance of exosomes in cardiovascular diseases is discussed below in three interconnected areas:

### 3.1. Pathophysiological Roles of Exosomes in Cardiovascular Diseases

#### 3.1.1. Myocardial Infarction and Ischemia–Reperfusion Injury

Cardiomyocytes under ischemic stress release exosomes rich in damage-associated proteins and stress-responsive RNAs, which can amplify inflammatory cascades. For example, exosomal miR-1 and miR-133a are elevated in the acute phase of myocardial infarction (MI) and contribute to cardiomyocyte apoptosis and arrhythmogenesis [1]. On the other hand, protective roles have also been identified: endothelial cell-derived exosomes carrying miR-126 promote angiogenesis and repair, while progenitor cell-derived vesicles reduce infarct size by regulating survival pathways [8,29].

#### 3.1.2. Heart Failure and Ventricular Remodeling

In chronic heart failure (HF), exosomes reflect the interaction between cardiomyocytes, fibroblasts, and immune cells. Fibroblast-derived exosomes rich in profibrotic factors, such as transforming growth factor-β (TGF-β)-related cargo, promote maladaptive remodeling, while cardiomyocyte-derived vesicles transport metabolic enzymes that reflect the energetic stress of the damaged myocardium [4]. Clinical studies indicate that circulating exosomal miRNAs, such as miR-423-5p and Long Intergenic Non-coding RNA Predicts CARdiac Remodeling (LIPCAR), are associated with adverse remodeling and poor outcomes, thus positioning them as potential prognostic biomarkers [40].

#### 3.1.3. Atherosclerosis and Vascular Dysfunction

Vascular cells secrete exosomes that contribute to atherogenesis. Endothelial exosomes carrying adhesion molecules (e.g., ICAM-1) promote leukocyte recruitment, while macrophage-derived vesicles rich in inflammatory mediators increase plaque instability [41]. Smooth muscle cell-derived exosomes transfer proliferation-associated RNA and proteins, promoting intimal hyperplasia. At the same time, endothelial progenitor-derived exosomes carrying proangiogenic cargo may exert stabilizing effects on plaques, suggesting context-dependent roles [4].

#### 3.1.4. Arrhythmias and Electrophysiological Remodeling

Emerging evidence links exosomal signaling to arrhythmia substrates. For example, ischemia-induced cardiomyocyte exosomes rich in miR-1 may predispose to conduction abnormalities by altering calcium handling and gap junction expression [3]. In atrial fibrillation, altered exosomal miRNA profiles have been associated with atrial fibrosis and electrical remodeling, but mechanistic studies are limited.

### 3.2. Biomarker Potential of Exosomes in Cardiovascular Diseases

One of the most promising applications of exosomes in cardiovascular medicine is their use as circulating biomarkers. Because exosomes encapsulate molecular cargo from their cells of origin and are dynamically released during disease processes, they provide a "liquid biopsy" of cardiovascular pathology. Compared to traditional biomarkers such as troponins or natriuretic peptides, exosomal signatures may provide greater molecular diversity and cell type-enriched information, and some candidates may exhibit early temporal variations; however, much of the evidence is exploratory and affected by pre-analytical variability and isolation methods [38,39].

#### 3.2.1. Acute Myocardial Infarction

Exosomal microRNAs (miRNAs) have shown strong potential as diagnostic markers in acute myocardial infarction (AMI). Studies have reported that cardiomyocyte-derived exosomal miR-1, miR-133a, miR-208a, and miR-499 levels increase in the early hours following infarction, paralleling cellular damage [1]. Temporal analyses of circulating (plasma) miR-208a demonstrate detectability within 1–4 h after coronary occlusion, supporting the broader concept that time-sensitive circulating RNAs may provide complementary early signals; however, this evidence does not represent a validated superiority over high-sensitivity troponin in clinical workflows [26]. Integrating such time-sensitive exosomal signals could significantly improve early triage and intervention strategies in AMI.

#### 3.2.2. Heart Failure

In chronic HF, circulating exosomes reflect maladaptive remodeling and metabolic stress. Exosomal miR-423-5p and the long non-coding RNA LIPCAR have been associated with ventricular remodeling and poor clinical outcomes [4]. Several studies have reported the functional roles of exosomal miR-21 and miR-30a in cardiovascular pathology. Exosomal miR-21 plays a role in the regulation of fibrosis and repair by regulating apoptosis, angiogenesis, and fibroblast activation through pathways such as PTEN/Akt [42,43]. In contrast, exosomal miR-30a has been shown to inhibit cardiomyocyte autophagy and worsen cardiac function after AMI [44].

These biomarkers could improve risk stratification and guide personalized treatment selection, particularly as HF phenotypes become increasingly heterogeneous. To clarify the difference between circulating and EV-validated biomarkers and to summarize the current evidence base, representative cardiovascular biomarkers with validated performance metrics and validation status are summarized in Table 1.

#### 3.2.3. Atherosclerosis and Coronary Artery Disease

Exosomes derived from endothelial cells, macrophages, and smooth muscle cells contribute to atherosclerotic progression and provide measurable circulatory signatures. For example, endothelial exosomes enriched in adhesion molecules such as ICAM-1 have been associated with plaque instability [41]. Macrophage-derived vesicles carrying miR-146a or inflammatory proteins are associated with lipid-rich, rupture-prone plaques [8]. Such signatures may complement imaging methods in identifying high-risk atherosclerotic lesions before clinical events.

#### 3.2.4. Arrhythmias

Although less studied, exosomal miRNAs are emerging as candidate biomarkers in arrhythmias. In atrial fibrillation (AF), changes in exosomal miR-328 and miR-21 levels have been associated with atrial fibrosis and electrical remodeling, correlating with disease severity [3]. These findings highlight the potential of exosomal profiling to identify substrates of AF recurrence after ablation or surgical correction.

### 3.3. Therapeutic Opportunities as Tools for Regenerative or Targeted Interventions

Exosomes have not only served as biomarkers but have also emerged as promising therapeutic agents and delivery vehicles in cardiovascular diseases. Their ability to encapsulate and protect nucleic acids, proteins, and lipids, combined with their low immunogenicity and stability in circulation, makes them attractive alternatives to synthetic nanoparticles [55].

Regenerative medicine: In myocardial infarction and heart failure, exosomes derived from stem/progenitor cells, endothelial cells, or cardiomyocytes have been shown to promote angiogenesis, reduce fibrosis, and increase cardiomyocyte survival. For example, cardiac progenitor cell-derived vesicles carrying pro-survival miRNAs reduce infarct size and improve left ventricular function in preclinical models [56,57]. Mesenchymal stem cell-derived exosomes are being tested in early-phase clinical trials for ischemic heart disease and offer encouraging safety profiles [58].

Targeted interventions: Advances in bioengineering now allow for the modification of exosomal membranes to improve tropism toward specific tissues, including damaged myocardium and atherosclerotic plaques. Strategies include surface peptide display, antibody conjugation, and hybrid exosome–liposome systems [59]. Engineered exosomes carrying therapeutic RNAs such as miR-126 or miR-210 have demonstrated improved cardiac repair in animal models [60]. Furthermore, exosome-mimicking nanovesicles produced by extrusion methods offer scalable alternatives with similar biodistribution and improved manufacturing feasibility [35].

Device integration: New approaches are exploring exosome-functionalized biomaterials, including drug-eluting stents and vascular grafts, to enable localized therapeutic delivery. In preclinical studies, incorporating exosomes into stent coatings has been shown to accelerate endothelialization and reduce restenosis [34]. This suggests that nanomedicine and interventional cardiology will converge in the future.

Table 2 summarizes the main preclinical and translational strategies investigating exosomes or exosome mimic systems as therapeutic tools in cardiovascular diseases, highlighting their mechanisms, applications, and limitations.

Translational challenges: Despite promising results, the therapeutic application of exosomes in cardiovascular diseases faces significant translational hurdles. Large-scale production and purification remain technically challenging, and differences in isolation methods lead to inconsistent vesicle yield and cargo composition [20]. Furthermore, patient-to-patient variability in exosome profiles complicates therapeutic standardization, and regulatory agencies have not yet established clear quality control criteria for exosome-based products. These challenges highlight the need for advanced analytical approaches that can integrate multi-omic cargo data, vesicle heterogeneity, and patient-specific variables. AI emerges as a particularly promising solution to these challenges by providing computational frameworks capable of addressing the multidimensional complexity found in exosomal therapy.

These translational challenges, combined with recent advances in computational biology, position AI as a key tool for addressing exosomal complexity. ML and DL approaches offer powerful frameworks for addressing the complexity of exosome biology. The following section provides an overview of AI approaches applied to exosomal biomarker discovery.

## 4. AI in Exosomal Biomarker Discovery

The complexity of exosomal biology, resulting from heterogeneous cellular origins, diverse molecular cargo, and patient-specific variability, creates analytical challenges that exceed the capabilities of traditional statistical methods. AI, encompassing machine learning (ML) and deep learning (DL), offers powerful approaches to extract patterns from high-dimensional datasets and identify clinically applicable biomarkers. By integrating transcriptomic, proteomic, lipidomic, and metabolomic signatures, AI frameworks can advance exosomal research beyond proof-of-concept studies to precision cardiovascular medicine [12,13].

### 4.1. Overview of AI Approaches

Exosomes contain thousands of RNA, protein, lipid, and metabolite species. This creates a high-dimensional and heterogeneous data environment that challenges traditional statistical methods [20,61]. In this context, AI offers a powerful toolset for analyzing complex exosomal datasets in CVDs.

AI is an umbrella term encompassing a wide range of technologies, ranging from ML to DL and network-based approaches. These approaches share the common goal of computationally simulating human intelligence. AI methods offer strategies for extracting disease-related signatures, improving diagnostic accuracy, and stratifying patients for prognosis or treatment [13,62].

ML, a subset of AI, uses a mathematical framework to make predictions by identifying distinctive patterns in data. DL, a subset of ML, uses multilayer neural networks to automatically learn hierarchical features from raw data, making it highly suitable for complex exosomal datasets [63]. In general, ML models such as support vector machines (SVMs) and random forests (RFs) are effective for feature selection and combinatorial biomarker panels [64,65]. DL architectures, such as convolutional neural networks (CNNs), recurrent neural networks (RNNs), and autoencoders, can capture nonlinear relationships and temporal dynamics. Network-based approaches, including graph neural networks (GNNs), extend these capabilities by integrating exosomal data with biological interaction networks and clinical metadata [66].

Applying AI to analyze large, high-dimensional datasets like multi-omics significantly increases the efficiency of mechanistic studies and clinical applications in cardiovascular research. This allows researchers to uncover subtle relationships that traditional methods might miss.

**Figure 2:**
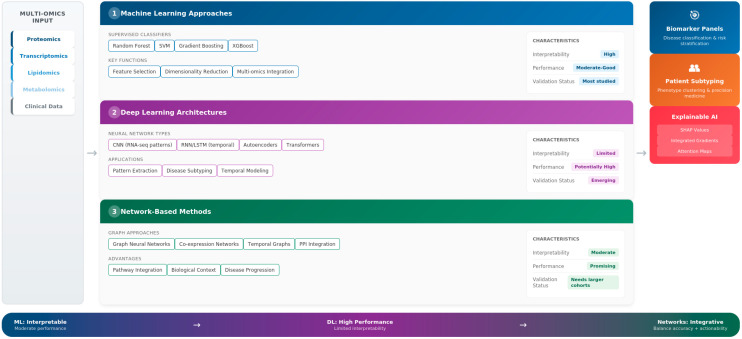
AI methodologies for exosome biomarker discovery in cardiovascular diseases. Overview of (**1**) machine learning approaches (RF, SVM, and ensemble methods), (**2**) deep learning architectures (CNN, RNN/LSTM, and autoencoders), and (**3**) network/graph-based methods with explainable AI frameworks. Reported performance varies depending on cohort size, validation design, and disease context; methodological considerations are discussed in Section 4 (and Table 3, where applicable). Created by the authors.

AI methodologies applied to exosomal biomarker discovery range from traditional machine learning to advanced DL and network-based approaches, each offering distinct advantages in addressing the complexity of cardiovascular exosomal data (Figure 2). These methods share the common goal of extracting clinically meaningful signatures from high-dimensional, heterogeneous datasets but differ fundamentally in their mechanisms, interpretability, and performance characteristics.

### 4.2. Machine Learning Applications

Wang et al. [26] reported the early detectability of circulating (plasma) miR-208a after coronary occlusion, supporting the broader concept that time-sensitive circulating RNAs may provide complementary early signals in AMI. However, this study did not specifically measure miR-208a in exosomes, and the current evidence does not support replacing high-sensitivity troponin in routine clinical workflows; rather, EV-associated signatures are exploratory and require standardized preliminary analyses and external validation.

Exosomal datasets typically contain thousands of variables per sample, many of which have small but additive effects. More recent applications have focused on integrating multiple biomarkers. ML classifiers can rank features by predictive importance, reduce dimensionality, and create panels that provide strong diagnostic accuracy. Zhou et al. [49] applied ML classifiers to plasma exosomal proteomes in AMI patients. Their study identified a panel of seven exosomal proteins (F13A1, TSPAN33, YWHAZ, ITGA2B, GP9, GP5, and PPIA) that distinguished AMI patients from controls, each exhibiting an area under the curve (AUC) >0.80.

A significant advance is the application of ML to multi-omics integration. By highlighting that ensemble models combining transcriptomic, proteomic, and clinical features consistently outperform single-omics classifiers, Lin et al. [13] examined three strategies, including early, mid-, and late integration. For example, ensemble learning improved stratification for secondary prevention of atherosclerotic cardiovascular diseases (ASCVDs) by improving the AUC from 0.75 to 0.81.

Representative applications are summarized in Table 3. In particular, pairing plasma exosomal proteomics with supervised ML distinguished AMI from controls [47], while multi-omics integration consistently outperformed single-omics approaches [13]. Graph-based and GNN models are already used in cardiovascular prediction [62] and exosome spectral classification [50]. However, their application to exosomal multi-omics in CVD remains an opportunity rather than the current standard practice.

### 4.3. Deep Learning and Network-Based Approaches

#### 4.3.1. Deep Learning for Feature Representation and Temporal Modeling

DL methods extend ML by capturing nonlinear and hierarchical relationships in exosomal data. CNNs applied to RNA sequencing data have identified complex miRNA expression patterns associated with myocardial remodeling and heart failure progression. For example, a hard voting ensemble (HVE) model trained with selected miRNAs achieved an accuracy of 0.86 and an AUC of 0.83 on an independent test set [67]. A specific variation of RNN, including long short-term memory (LSTM) architectures, was particularly well-suited to longitudinal exosomal data [68]. These kinds of observations make them a valuable strategy for monitoring biomarker dynamics during post-AMI recovery.

Unsupervised learning, particularly clustering, plays a crucial role in disease subtyping and patient classification. Due to the abundance of large-scale multi-omics data, including exosomal omics data, DL models such as autoencoders can enhance clustering algorithms by exploiting inter-individual heterogeneity [69]. While direct cardiovascular applications remain limited, early studies suggest that autoencoder-derived clusters can better capture disease heterogeneity than traditional unsupervised methods.

#### 4.3.2. Network-Based and Graph Neural Network Approaches

Beyond the unimodal approaches, network-based strategies offer complementary insights. Graph neural networks (GNNs) and co-expression network analyses integrate exosomal features with protein–protein interactions, signaling networks, and clinical variables. In cardiovascular contexts, network medicine approaches have revealed modules linked to endothelial dysfunction and vascular inflammation [12,62].

Spectral graph methods have also been tested for exosome classification using Raman spectroscopy data. Ngo et al. [50] demonstrated the feasibility of graph-based classifiers applied to exosome Raman spectra, achieving accuracies ranging from 0.76 to 0.86, depending on preprocessing. This proof-of-concept highlights how graph architectures can handle exosome-specific features beyond traditional omics.

#### 4.3.3. Explainable AI and Clinical Interpretability

Importantly, DL and GNN models are increasingly paired with explainable AI (XAI) frameworks such as SHAP (SHapley Additive Descriptions) and integrated gradients [70]. This helps clarify which exosomal features drive predictions. The interpretability is critical for clinical confidence and regulatory acceptance. It ensures that complex models provide not only accuracy but also transparency for clinical decision-making.

These explainability methods support transparency and clinical interpretability in AI-powered decision support systems. Explainable AI approaches such as SHAP and gradient-based attribution make model outputs more interpretable and verifiable, enabling clinicians to better understand predicted risk factors and support safe clinical use [70]. From a workflow perspective, attribution visualizations facilitate collaborative decision-making between clinicians and patients by highlighting which EV characteristics most strongly influence predictions. For example, an explanatory SHAP-based interface might show that an AMI risk prediction is primarily driven by high exosomal miR-208a and low miR-126, with demographic factors contributing minimally. Such transparent attribution is increasingly required to support clinician trust, enable meaningful informed consent, and meet emerging expectations regarding the adoption of AI-powered clinical decision support systems by regulatory bodies.

From supervised machine learning classifiers to deep neural networks and graph-based integration, these AI approaches collectively show significant potential for advancing exosomal biomarker discovery in cardiovascular medicine (Figure 2). However, despite these encouraging developments, several limitations remain that limit the clinical application of AI-assisted exosomal biomarker discovery.

### 4.4. Limitations of Current AI Approaches in Exosomal Research

Despite encouraging advances, several limitations constrain the clinical application of AI-assisted exosomal biomarker discovery.

First, existing studies are often dominated by small and heterogeneous cohorts of fewer than a few hundred patients, limiting statistical power and model generalizability [49,50]. Second, while few AI studies explicitly control for pre-analytical variables [20], different exosome isolation and characterization methods produce heterogeneous datasets. This makes model generalization across studies and institutions difficult. Thirdly, most models are trained and tested within a single dataset, and a lack of external validation is common. A relevant and often underestimated concern is the increasing problem of overfitting in EV biomarker studies due to feature spaces being extremely large (*p* ≫ *n*) while sample sizes are often small. Without strict separation of training/validation/test data, feature selection performed outside cross-validation, or repeated peeking at outcomes during pipeline tuning, reported performance can be inflated by data leakage [71]. Leakage can occur subtly through batch effects associated with case–control cases, through normalization steps applied to the entire dataset before splitting, or through repeated model selection on the same test set. To mitigate these risks, EV-focused AI studies should adopt nested cross-validation for feature selection and hyperparameter tuning, maintain an untouched holdout set whenever possible, and prioritize external validation across sites, platforms, and isolation workflows. Where multicenter external datasets are not available, a practical minimum requirement is temporal validation (e.g., later recruitment periods) and rigorous reporting of preprocessing, missing data handling, and batch correction decisions. Finally, transparent reporting frameworks for predictive modeling and clinical AI assessment (e.g., TRIPOD+AI, PROBAST+AI, and DECIDE-AI) can help standardize documentation of cohorts, endpoints, and analytical pipelines, improving reproducibility and interpretability [72,73,74].

This particularly impacts the robustness of multi-omic models. Furthermore, many AI models operate as "black boxes," providing high accuracy without sufficient interpretability. While frameworks such as SHAP increase transparency [70], their use in exosomal studies remains limited.

Another limitation stems from the temporal dynamics of exosomes. The dynamic nature of their release requires sophisticated modeling approaches that can capture temporal relationships and patterns of disease progression [26]. Finally, integration with clinical practice is lacking. Translating complex AI models into clinical workflows requires user-friendly interfaces, standardized protocols, and integration with existing healthcare systems. However, few studies have compared AI-based exosomal classifiers with established cardiovascular biomarkers such as troponin or NT-proBNP [75].

**Figure 3:**
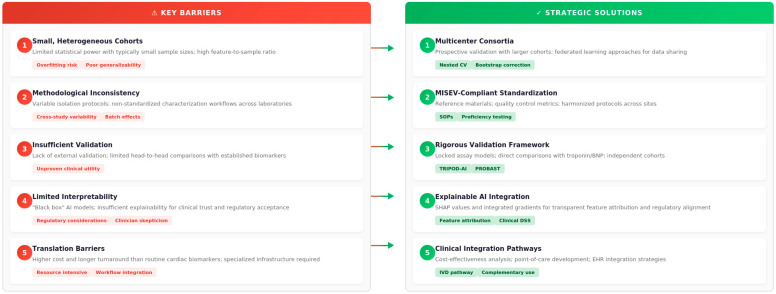
Limitations and solutions of AI-assisted exosomal biomarker discovery. Left panel: Key barriers to clinical implementation. Right panel: Corresponding strategies for improving reproducibility, validation rigor, and clinical integration. Detailed discussion is in Section 4.4 and Section 6. Created by the authors.

All these limitations highlight the need for larger, multicenter cohorts, standardized EV workflows, transparent modeling practices, and comparison with gold-standard biomarkers to translate AI-assisted exosomal research into clinical practice. These challenges and potential solutions are systematically addressed in Figure 3, which provides a comprehensive framework outlining the key barriers hindering clinical translation and the evidence-based strategies needed to overcome each barrier. This structured approach emphasizes that successfully integrating AI-based exosomal classifiers into cardiovascular medicine requires coordinated efforts encompassing technical standardization, methodological rigor, computational transparency, and regulatory compliance.

#### 4.4.1. Small *n*, Large *p* Effects and Overfitting Risks

A critical methodological problem in current AI-powered exosomal research is the ‘small *n*, big *p*’ problem, where the number of features (>31,000 potential cargo molecules) significantly exceeds the sample size (typically *n* < 50). This imbalance creates a feature-to-sample ratio exceeding 600:1 and virtually guarantees spurious correlations if proper adjustment is not made.

#### 4.4.2. Validation Design, Data Leakage, and Reproducibility

Specific overfitting patterns observed in exosomal AI studies include: (1) feature selection performed on the entire dataset before cross-validation splitting, leading to information leakage; (2) bulk effect correction using test set information; (3) hyperparameter tuning without nested validation; and (4) reporting training set performance without proper retention testing. Studies show that data leakage and non-nested model selection can substantially inflate apparent discrimination performance, especially in small datasets [71]. Proposed solutions include: (i) nested cross-validation with outer loops for performance estimation and inner loops for hyperparameter tuning; (ii) bootstrap optimism correction to correct for overfitting; (iii) permutation testing to generate null distributions for trait significance; (iv) minimum sample size calculations following the ‘10 events per variable rule’; and (v) mandatory external validation in independent cohorts. Compliance with TRIPOD-AI guidelines ensures transparent reporting of model development and validation procedures.

### 4.5. Exosomes vs. Established Cardiac Biomarkers

Despite the promise of exosomal biomarkers, traditional cardiac markers such as troponins and BNP remain the clinical gold standards. High-sensitivity troponin assays detect myocardial injury within hours of symptom onset with over 95% sensitivity and specificity, supported by extensive multicenter validations and standardized cutoff values [76,77]. BNP and NT-proBNP are similarly established in the diagnosis and monitoring of heart failure and have strong prognostic value in diverse populations. A contextual comparison with established cardiac biomarkers highlights both the potential and current limitations of EV-based and circulating molecular signatures (Table 4).

In contrast, exosome-derived signatures (whether miRNAs, lncRNAs, or proteomic profiles) are still in early-stage proof-of-concept status. As seen in Table 2, studies generally involve small cohorts (*n* < 50 in validation), heterogeneous isolation methods, and variable bioinformatics processes. Reported AUC values (>0.80) are encouraging but lack the consistency and reproducibility required for regulatory acceptance [49]. Furthermore, pre-assay variability in exosome isolation (e.g., ultracentrifugation and size exclusion) contributes to significant inter-study differences rarely seen in standard troponin assays.

From a practical perspective, clinicians are unlikely to replace troponin or BNP based on the existing data. Because the exosome assays remain relevant, they would need to demonstrate: (1) equivalent or superior sensitivity/specificity in large prospective cohorts, (2) rapid turnaround compatible with emergency workflows, and (3) cost-effectiveness compared to existing immunoassays. Currently, exosomes appear best positioned as complementary biomarkers.

Looking ahead, AI carries the potential to bridge this gap. By integrating heterogeneous datasets across multi-omic exosome studies, AI-driven models can reduce variability, identify powerful combinatorial signatures, and highlight clinical contexts. In this sense, AI methodologies accelerate exosome-based biomarkers to move from proof-of-concept to implementing in practice.

## 5. Artificial Intelligence-Assisted Exosome-Based Therapies

### 5.1. Exosome-Based Regenerative Therapies

Exosomes derived from mesenchymal stem cells (MSCs), endothelial progenitor cells, and cardiac progenitor cells have demonstrated regenerative potential in models of myocardial infarction, ischemia–reperfusion injury, and heart failure. These vesicles carry cardioprotective payloads such as miR-21, miR-126, and miR-210, which regulate apoptosis, angiogenesis, and fibrosis pathways [29,56,57]. In preclinical studies, MSC-derived exosomes improved ventricular remodeling and contractile recovery after myocardial infarction, with effects comparable to parental cells but a more favorable safety profile [34,58].

However, clinical translation has been hampered by patient-to-patient variation in exosome composition and uncertainties in predicting therapeutic response. AI offers new opportunities in this regard. By integrating exosomal multi-omics with patient-level clinical data, ML models can classify potential responders from non-responders, optimize dosing regimens, and even predict long-term outcomes of regenerative therapy [12,68]. For example, clustering algorithms applied to exosomal miRNA signatures can identify subgroups of heart failure patients most likely to benefit from vesicle-based interventions.

### 5.2. Artificial Intelligence for Exosome Engineering

Beyond predictive response modeling, AI is increasingly being used in the design and engineering of therapeutic exosomes. Computational frameworks can simulate cargo loading efficiency, optimize the integration of cardioprotective miRNAs, and even predict the efficacy of CRISPR-based genome editing components packaged within vesicles [19]. Deep learning models that analyze sequence–structure relationships enable rational selection of miRNAs or lncRNAs most likely to achieve therapeutic efficacy while minimizing off-target effects.

AI can also support the optimization of delivery and biodistribution. Predictive pharmacokinetic modeling based on imaging and biodistribution datasets allows for the refinement of surface modifications (e.g., peptide targeting, membrane fusion strategies) to enhance cardiac tropism [35,68]. Similarly, reinforcement learning-based simulations have been proposed to iteratively test combinations of vesicle modifications and predict their functional outcomes in silico before costly laboratory validation. However, most AI-powered exosome therapies are still in the preclinical stage, with only natural exosome preparations having entered early-stage clinical trials.

**Figure 4:**
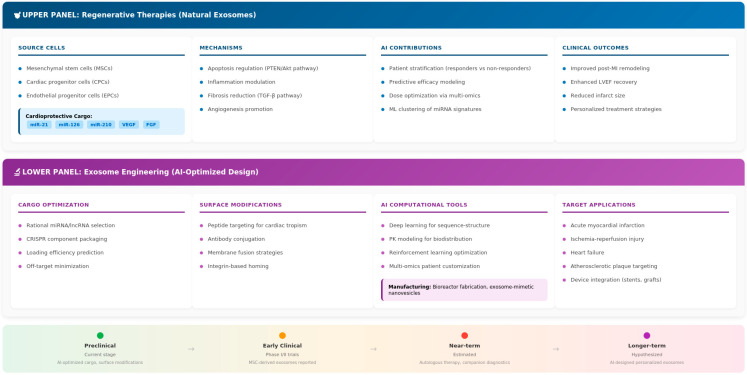
AI-powered exosome-based therapies for cardiovascular diseases. Top panel: Regenerative therapies using naturally occurring stem cell-derived exosomes with AI-guided patient classification and dose optimization. Lower panel: AI-assisted exosome engineering for cargo optimization, surface modification, and scalable manufacturing. Translation timeline is illustrative. Clinical applications and development stages are discussed in Section 5 and Table 2. Created by the authors.

Taken together, the integration of AI is transforming therapeutic exosomes from experimental tools into programmable, precision platforms. By combining regenerative biology with predictive modeling, these approaches can accelerate the development of clinically effective exosome-based therapies for cardiovascular diseases. To illustrate these dual trajectories, Figure 4 provides a comprehensive framework illustrating how AI contributes to both natural exosome-based regenerative therapies and engineered exosome design. The figure highlights the critical role of AI in optimizing therapeutic cargo selection, improving delivery strategies, predicting patient responses, and ultimately enabling precision cardiovascular applications that address the complexity and heterogeneity inherent in exosomal therapies.

## 6. Limitations, Challenges, and Future Directions

Despite significant progress in exosome research and the integration of AI, several challenges continue to limit clinical translation. These limitations span technical, biological, computational, and regulatory domains. The following subsections reflect the multifaceted obstacles to clinical application, separating technical and computational constraints from biological uncertainty, economic feasibility, and regulatory considerations.

### 6.1. Standardization and Reproducibility

In EV biomarker and therapeutic studies, the dominant obstacle to transitioning to clinical practice is reproducibility, stemming primarily from heterogeneity in experimental workflows rather than the inherent limitations of artificial intelligence. Differences in pre-analytical procedures (collection tubes, anticoagulant selection, processing time, centrifugation, storage conditions, and freeze–thaw cycles), isolation approaches (ultracentrifugation, size exclusion chromatography, sedimentation, immunoaffinity capture, or microfluidics), and characterization/reading methods can produce EV preparations that are not directly comparable between laboratories, which limits reproducibility and makes inter-study synthesis difficult [82]. Consequently, candidate signatures identified in small cohorts may reflect method-specific artifacts or differences in sample processing, and models trained in one institutional pipeline often fail to generalize when applied to independent datasets.

For AI-powered EV biomarkers, reproducibility should be considered a prerequisite, not an afterthought. Practical measures include adherence to MISEV-compliant reporting, clear operational definitions (e.g., "EVs smaller than 200 nm" when biogenesis cannot be proven), standardized quality controls for lipoproteins and hemolysis, and transparent documentation of batch effects and normalization strategies. Multicenter studies should pre-define and harmonize protocols, include reference materials and interlaboratory comparisons whenever possible, and lock down analytical processes prior to validation. Without these steps, even technically advanced models (e.g., deep learning and graph-based methods) risk learning site-specific or workflow-specific patterns instead of disease biology, limiting both clinical confidence and regulatory readiness.

### 6.2. Pre-Analytical Variability and Its Impact on AI Model Reliability

Pre-analytical variability represents a significant source of latent bias in EV-based biomarker studies and directly affects the reliability and generalizability of AI models. Factors such as collection tube material and anticoagulant selection, venous puncture technique, processing time, centrifugation parameters, storage temperature and duration, and number of freeze–thaw cycles can significantly alter EV concentration, size distribution, surface marker expression, and apparent molecular load, thus limiting reproducibility across studies and laboratories [20,29]. In blood-derived samples, additional confounding factors, including lipoprotein co-isolation, hemolysis, and platelet activation, can introduce non-vesicular nucleic acids and proteins that distort downstream omics profiles and inflate the apparent signal, particularly in small cohorts when isolation and characterization workflows are not aligned [30,82].

From a computational perspective, unmeasured pre-analytical variables increase the risk of AI models learning workflow-specific or batch-specific signatures instead of disease biology, contributing to optimistic internal performance but poor inter-study generalization [74]. This effect is particularly problematic for high-dimensional multi-omics datasets where subtle systematic biases can dominate feature selection and compromise external validation [57,58]. Consequently, rigorous documentation and harmonization of pre-analytic workflows, implementation of quality control measures (e.g., hemolysis indices and lipoprotein-related markers), and transparent reporting of sample processing protocols should be considered prerequisites for developing reliable AI in EV research [30]. Where harmonization is not possible, analytical strategies such as batch-sensitive modeling, sensitivity analyses, and layered validation should be explicitly reported to contextualize model performance and uncertainty [49,50].

### 6.3. Cost-Effectiveness and Infrastructure

Clinical application also depends on cost and scalability. Current isolation methods are labor-intensive and expensive, requiring ultracentrifugation or advanced microfluidics, limiting their applicability outside specialized centers. Developments such as microfluidic devices and bioreactor-based large-scale vesicle production hold promise for reducing costs and increasing yields. However, these technologies are not yet routinely available, and the current exosome isolation and analysis methods are significantly more expensive than traditional biomarker assays. Chae et al. [83] indicate that exosome therapies may eventually become cost-effective, but this depends on resolving manufacturing and distribution bottlenecks that remain unresolved.

### 6.4. Computational and Technical Bottlenecks

AI-based models require clean, high-quality input data. However, kit-based extraction methods, while faster and more clinically applicable, often isolate protein aggregates and lipoproteins together, reducing data quality. These impurities compromise downstream molecular analyses and can impact ML models that assume uniformly processed input [49].

### 6.5. Clinical and Regulatory Considerations

From a translational perspective, exosome-based diagnostic and therapeutic methods face significant regulatory hurdles. Translating AI-developed exosomal biomarkers into clinical workflows requires standardization not only in laboratory procedures but also in computational processes [31,84]. Few exosome-derived products have progressed beyond phase I or II clinical trials, and issues of GMP-compliant manufacturing, pharmacokinetics, and long-term safety profiles have not yet been fully addressed. Regulatory bodies have not yet defined clear approval pathways for exosome products, creating uncertainty for both researchers and industry developers [85].

Addressing these gaps and achieving the translation will depend on several key issues: (1) large scale, prospective validation studies; (2) head-to-head comparisons with troponin/BNP for different conditions such as AMI and HF; (3) establishing consensus regulatory frameworks (e.g., clear FDA/EMA guidance on exosome-based diagnostics covering manufacturing, quality control, and clinical validation); (4) embedding AI-exosome tools into electronic health records (EHRs).

### 6.6. Future Directions

The next decade of AI-assisted exosomal biomarker discovery will likely proceed in phased steps. Short-term (2–5 years) studies should focus on methodological consistency, including stricter adherence to MISEV guidelines, the development of reference standards, and consistent isolation protocols. Explainable AI (XAI) approaches that increase clinical confidence, as well as large multicenter cohorts with diverse populations, are also important.

In the medium term (5–10 years), the emphasis will shift to clinical validation. Randomized trials comparing AI-assisted exosomal biomarkers to troponin and natriuretic peptides will be critical for generating added value. Parallel developments in point-of-care EV analysis and automated analysis processes may enable initial implementation in specialized cardiovascular centers.

In the longer term (>10 years), integration into precision medicine could include dynamic risk prediction, treatment monitoring, and even treatment guidance. However, given the persistent challenges of reproducibility, cost-effectiveness, and regulatory clarity, widespread adoption remains a distant goal.

Accelerating this trajectory requires coordinated efforts across experimental, computational, and clinical domains. Researchers in the experimental domain should emphasize standardization and reproducibility, while funding agencies in the computational domain should invest in infrastructure and collaboration networks, and clinicians in the clinical domains should ensure clinical relevance and guard against premature translation.

## 7. Conclusions

The convergence of exosome biology and artificial intelligence is reshaping perspectives in cardiovascular research. While initial studies suggest that AI can uncover complex exosomal signatures with potential diagnostic and therapeutic value, these efforts remain largely limited to exploratory, proof-of-concept research. The transition to routine clinical care is limited by heterogeneous isolation methods, modest cohort sizes, and the lack of validated benchmarks against established biomarkers such as troponin or natriuretic peptides.

Nevertheless, this field holds real long-term promise. Advances in multi-omics integration, federated learning, and explainable AI could provide more reproducible and clinically interpretable models. Achieving this vision will require standardized methodologies, adequately powered validation studies in diverse populations, and close alignment with regulatory frameworks.

Rather than exaggerating the preparedness, it is important to recognize that the path to clinical application will be measured in decades, not years. However, with continued interdisciplinary collaboration and targeted investments in infrastructure, AI-powered exosomal biomarkers could become powerful tools for precision cardiovascular medicine, providing insights beyond the scope of current diagnostic methods.

Key Takeaways

- EV/exosome multi-omics datasets are highly dimensional and have batch sensitivity; reproducibility depends on pre-analysis and isolation workflows as well as AI model selection.
- Most reported EV biomarker classifiers are in the discovery phase (small cohorts, limited external validation); direct comparison with troponin/NT-proBNP is rare and should be prioritized.
- For small sample sizes, pipelines that account for leakage (nested cross-validation, strict preprocessing separation, and preferably external/temporal validation) are essential.
- While ensemble machine learning methods generally provide strong baseline performance with better interpretability, deep learning and graph-based models can be valuable for pathway-oriented integration but require larger cohorts.
- Multi-omics integration should be explicitly planned (early/mid/late fusion) and evaluated using consistent validation and reporting standards.
- MISEV-compliant EV reporting + transparent AI reporting frameworks (e.g., TRIPOD+AI/PROBAST+AI) improve interpretability and inter-study comparability. Readiness for translation depends not only on AUC but also on test lock-in, multicenter validation, workflow feasibility, and clinically significant endpoints.
- Short-term clinical impact is more likely in adjunctive risk stratification or phenotyping rather than replacing established biomarkers.

## Figures and Tables

**Table 1:** Circulating and EV-derived biomarkers in major cardiovascular diseases.

CVD Type	Biomarker(s)	Sample Source/EV Type	Validation Status	Reported Performance	References
AMI	miR-208a	Circulating plasma (not exosome-specific)	Multiple cohorts; meta-analysis available	Sensitivity 83%, Specificity 97%, AUC 0.93 (meta-analysis, *n* = 1703 AMI + 1589 controls)	[45,46]
AMI	miR-1, miR-133a, miR-499	Circulating plasma (muscle-enriched, not heart-specific)	Multiple small cohorts; meta-analysis pooled	miR-1: AUC 0.84; miR-499: AUC 0.91 (meta-analysis)	[47]
AMI	Exosomal miR-1915-3p, miR-4507, miR-3656	Serum exosomes	Exploratory; small cohort; AMI vs. SCAD comparison	miR-1915-3p AUC 0.772; miR-3656 AUC 0.771	[48]
AMI	7-protein panel (F13A1, TSPAN33, YWHAZ, ITGA2B, GP9, GP5, PPIA)	Plasma exosomes	Discovery (*n* = 20) + PRM validation (*n* = 21); single-center	All 7 proteins: AUC > 0.80 each	[49]
AMI	ML-derived miRNA panel (10 miRNAs via LASSO)	Circulating plasm (not exosome-specific)	GEO datasets; independent test validation	HVE model: Accuracy 0.86, AUC 0.83 on independent test set	[50]
HF	miR-423-5p	Circulating plasma	*n* = 50 dyspnea patients (30 HF) + 39 controls; single-center	AUC 0.91 for HF vs. healthy + non-HF dyspnea	[51]
HF	Exo-miR-92b-5p	Serum exosomes	*n* = 28 HFrEF + 30 controls; single-center	Sensitivity 71.4%, Specificity 83.3% for HFrEF	[52]
HF (Post-MI)	LIPCAR (lncRNA)	Plasma + EVs (transport via large EVs)	788 patients across 3 cohorts; prognostic value validated	Predicts LV remodeling; associated with CV death in chronic HF	[53,54]
AF	miR-328, miR-21	Circulating (not confirmed exosomal)		Associated with atrial fibrosis; limited diagnostic data	[3]
CAD	ICAM-1+ EVs	Endothelial-derived EVs		Associated with plaque instability (no AUC reported)	[41]

The validation status reflects whether the models were evaluated using internal cross-validation, separate testing, or independent external cohorts. Performance metrics are reported as described in the original studies. Note: Confirmed EV isolation demonstrates enrichment and characterization (e.g., particle sizing plus EV markers and/or imaging) consistent with MISEV recommendations. The term ‘in circulation’ refers to plasma or serum measurements without EV-specific isolation.

**Table 2:** Therapeutic exosome strategies in cardiovascular diseases.

Strategy	Application/CVD	Source/Engineering	Cargo Mechanism	Evidence Stage	Key References
Natural exosomes	Reducing infarct size, improving left ventricular ejection fraction, increasing angiogenesis	Cardiac progenitor cells, MSCs, endothelial cells	Rich in pro-survival and pro-angiogenic miRNAs (e.g., miR-21, miR-210) and growth factors	Preclinical (rodent, pig); early clinical studies ongoing	[46,47,48]
Engineered exosomes	Enhanced cardiac repair, reduced fibrosis, and promoting vascular regeneration	Surface peptide modification, antibody conjugation, RNA loading	Targeted delivery of therapeutic RNAs (e.g., miR-126, miR-210); improved tropism to myocardium or plaques	Preclinical animal models	[34,45]
Exosome-mimicking nanovesicles	Drug/RNA delivery to ischemic myocardium and atherosclerotic lesions	Produced by extrusion of cells; scalable manufacturing	Similar membrane proteins, customizable cargo; higher efficiency compared to native exosomes	Preclinical animal models	[35]
Device-integrated exosomes	Drug-eluting stent alternatives; vascular graft healing	Stent or graft coatings with exosomes or mimetics	Promotes re-endothelialization, prevents neointimal hyperplasia, local sustained delivery	Preclinical (in vivo stent models)	[34]

The evidence phase reflects the highest level of support currently available (preclinical in vitro/in vivo studies and early-stage clinical trials).

**Table 3:** Artificial intelligence applications in exosomal biomarker discovery for cardiovascular diseases.

Study	CVD Context	Data Type (EV/Exosome)	AI/Model	Cohort and Validation	Reported Performance	Notes
[49] [Zhou et al., 2025]	AMI (STEMI, NSTEMI, UA)	Plasma exosomal proteomics (label-free MS, DRM validation)	Statistical + machine learning classifiers (ROC, feature selection)	Discovery *n* = 20; Validation *n* = 21	ROC AUC > 0.80 for 7 proteins (F13A1, TSPAN33, YWHAZ, ITGA2B, GP9, GP5, and PPIA)	First validated exosomal protein biomarker panel for MI using ML analysis
[13] [Lin et al., 2025]	Mixed CVD outcomes	Multi-omics (not EV-specific; transcriptomics + proteomics + clinical)	Stacking (Ensemble ML: RF, SVM, GBM)	Multi-cohort integration; cross-validation	Improving AUC from 0.75 to 0.81 compared to the traditional risk score, enabling risk stratification for secondary prevention of ASCVDs	Not exosome-specific, but demonstrates integration strength with EV data
[50] [Ngo et al., 2025]	General exosome classification (not disease-specific)	Exosome Raman spectra	Graph-based + tree classifiers	Benchmark dataset; internal cross-validation	Reported accuracy (varies by classifier, ~80–90%)	Proof of concept for graph spectral AI in exosome classification
[62] [Lundström et al., 2023]	ASCVD progression	Clinical EHR + LDL trajectories (not EV-specific)	Explainable GNNs (temporal graph networks)	Early-stage project, retrospective	Shows the applicability of GNN for ASCVD modeling	Supports future integration of EV multi-omics with GNN frameworks

Validation is reported as described in the original studies (e.g., cross-validation, holdout test set, temporal validation, or external validation). Performance metrics are not directly comparable between studies due to differences in cohorts, EV workflows, and endpoints.

**Table 4:** Comparison with established cardiac markers.

Parameters	hs-Troponin (AMI)	NT-proBNP/BNP (HF)	Exosomal/Circulating Biomarkers
Primary indication	AMI Diagnosis (Rule-in/Rule-out/Rejection)	Acute heart failure diagnosis; chronic heart failure prognosis	Exploratory (AMI, HF, CAD, and AF)
Sensitivity	90–99% (0/1 h or 0/3-h algorithms)	90–96% (exclusion at <300 pg/mL)	71–86% (study dependent)
Specificity	74–96% (depending on threshold value)	65–84% (age-adjusted)	75–97% (limited validation)
AUC	0.94–0.96	0.85–0.93	0.77–0.93
Sample size (typical)	>10,000 patients in validation studies	>1000 patients in validation studies	<50 patients typical; largest *n* = 788
External validation	Multiple independent cohorts, multicenter RCTs	Multiple independent cohorts	Rarely; mostly single-center
Standardization	FDA-approved tests; 99th percentile determined	Age-classified cutoffs; FDA-approved	No consensus; MISEV guidelines for isolation only
Regulatory status	FDA-approved; Class I recommendation	FDA-approved; Class I recommendation	Unapproved; no defined pathway
Turnaround time	15–60 min (POC available)	15–60 min (POC available)	4–8+ h (research only)
Approximate cost	$10–30 per test	$15–50 per test	$100–$500+ (research setting)
Key limitation	Increases in non-cardiac conditions (renal failure, sepsis)	Age, obesity, and renal function levels affect the outcome	Pre-analytical variability; lack of standardization
References	[78,79]	[80,81]	[49,52,53,54]

Note: Costs vary depending on the platform, trading volume, and regional pricing.

## Data Availability

All supporting data are included in the article.
